# Designing a Dyad-Based Digital Health Intervention to Reduce Sedentary Time in Black Breast Cancer Survivors and Their First-degree Relatives: Human-Centered Design Study

**DOI:** 10.2196/43592

**Published:** 2023-05-24

**Authors:** Meghan Blazey, Catherine Marinac, Jessica Whiteley, Sarah Peterson, Karen Burns White, Cathyanah Jacques, Helen Lam, Barbara Halpenny, Shree Patel, Raymond Lamothe, Julie Wright

**Affiliations:** 1 School of Nursing University of Rochester Rochester, NY United States; 2 Division of Population Sciences Dana-Farber Cancer Institute Boston, MA United States; 3 University of Massachusetts Boston Boston, MA United States; 4 Massachusetts College of Pharmacy and Health Sciences Boston, MA United States

**Keywords:** sedentary behavior, digital health, dyad-based intervention, breast cancer risk, breast cancer survivor, social support, mobile phone

## Abstract

**Background:**

Breast cancer, the most commonly diagnosed cancer and second leading cause of cancer-related death in women in the United States, disproportionately affects women from minoritized or low socioeconomic backgrounds. The average woman has an approximately 12% lifetime risk of developing breast cancer. Lifetime risk nearly doubles if a woman has a first-degree relative with breast cancer, and the risk increases as multiple family members are affected. Decreasing sedentary behaviors through moving more and sitting less reduces breast cancer risk and improves outcomes for cancer survivors and healthy adults. Digital health solutions, such as mobile apps that are culturally appropriate, designed with input from the target audience, and include social support, are effective at improving health behaviors.

**Objective:**

This study aimed to develop and evaluate the usability and acceptability of a prototype app designed with a human-centered approach to promote moving more and sitting less in Black breast cancer survivors and their first-degree relatives (parent, child, or sibling).

**Methods:**

This 3-phase study consisted of app development, user testing, and evaluation of user engagement and usability. Key community stakeholders were engaged in the first 2 (qualitative) phases to provide input into developing the prototype app (MoveTogether). After development and user testing, a usability pilot was conducted. Participants were adult breast cancer survivors who identified as Black and agreed to participate with a relative. Participants used the app and a step-tracking watch for 4 weeks. App components included goal setting and reporting, reminders, dyad messaging, and educational resources. Usability and acceptability were assessed with a questionnaire that included the System Usability Scale (SUS) and semistructured interviews. Data were analyzed with descriptive statistics and content analysis.

**Results:**

Participants in the usability pilot (n=10) were aged 30 to 50 years (6/10, 60%), not married (8/10, 80%), and college graduates (5/10, 50%). The app was used on average 20.2 (SD 8.9) out of 28 days—SUS score of 72 (range 55-95)—and 70% (7/10) agreed that the app was acceptable, helpful, and gave them new ideas. Additionally, 90% (9/10) found the dyad component helpful and would recommend the app to friends. Qualitative findings suggest that the goal-setting feature was helpful and that the dyad partner (*buddy*) provided accountability. Participants were neutral regarding the cultural appropriateness of the app.

**Conclusions:**

The MoveTogether app and related components were acceptable for promoting moving more in dyads of breast cancer survivors and their first-degree relatives. The human-centered approach, which involved engaging community members in the development, is a model for future technology development work. Future work should be done to further develop the intervention based on the findings and then test its efficacy to improve sedentary behavior while considering culturally informed strategies for adoption and implementation within the community.

**Trial Registration:**

ClinicalTrials.gov NCT05011279; https://clinicaltrials.gov/ct2/show/NCT05011279

## Introduction

### Background

Breast cancer, the most commonly diagnosed cancer and the second leading cause of cancer-related death in women in the United States, disproportionately affects women from minoritized and low socioeconomic backgrounds [1]. The average woman has an approximately 12% lifetime risk of developing breast cancer. Lifetime risk nearly doubles to approximately 24% if a woman has a first-degree relative with breast cancer, and the risk increases as multiple family members are affected [2]. For women who are Black, Latinx, or socioeconomically disadvantaged, mortality from breast cancer is up to 54% higher than mortality in non-Hispanic White women [1]. In addition, the incidence of breast cancer is increasing annually for women who are Black or Latinx, whereas the incidence of breast cancer in non-Hispanic White women remains stable [2]. Certain types of breast cancers are considered high risk, such as those that occur at a young age (<50 years) and those that have cancer with specific pathology (eg, triple-negative breast cancer). These high-risk diagnoses are more common and have more disparate outcomes in Black women than in all other groups [3]. Therefore, breast cancer survivors and those who are first-degree relatives of survivors are considered to be at high risk for breast cancer and could benefit from intervention.

On the basis of the poor outcomes from breast cancer diagnoses in Black women and the risk associated with having a first-degree relative diagnosed with breast cancer, research efforts must focus on cancer prevention within this domain. Addressing increased movement is one avenue that may have benefits for both the cancer survivor and relative considered at high risk. Any decrease in sedentary time leads to an increased preventive benefit [4], and the positive effect of physical activity (PA) has been noted even in the context of genetic or familial risk for breast cancer [5]. Although increasing PA reduces cancer risk, with some studies reporting a reduction of 10% to 39% [5], there are emerging data to suggest that higher rates of sedentary time, independent of PA or BMI [6], are associated with increased cancer risk. Specifically, recent data suggest that a high total time sitting was associated with a higher risk for breast cancer of 27% to 28%, independent of PA, and associations were stronger for receptor-negative tumors, which are considered high risk [7].

It has been observed that Black women interested in digital health interventions will engage in lifestyle interventions [8], especially if these interventions are designed to be culturally appropriate [9,10]. An advantage of using a digital health approach, such as a smartphone app, is the ability to provide tailored messages for diverse groups of adults [11]. However, a consistent finding is that many commercially available apps *do not use* evidence-based behavior change techniques [12], are not informed by theory [13], and are not rigorously evaluated [13,14]. A review study found 185 available apps relevant to breast cancer care and management, but only 10% involved medical experts in their creation, and only 11% were evidence based [15]. Alarmingly, the review found that 15.7% of the apps had the potential to cause harm owing to a lack of evidence-based development. Therefore, potential translational public health benefits are missed.

In summary, there is a need for rigorously developed and tested theory-based digital health interventions for cancer prevention. Leveraging design strategies that place user input at the center of development and testing of the interventions is critical for developing effective interventions [16]. This process, called human-centered design [17-19], is imperative to the success of digital health apps for health behavior change.

We applied social cognitive theory (SCT) [20] to design the app in this study. SCT posits that behavior is influenced by a 3-way reciprocal interaction among the environment, the individual or person, and behavior. Social support is a known mediator of PA in several studies and formed the basis for creating a family-based intervention. Social support is also a known contributor to the success of PA-based interventions in Black breast cancer survivors [21,22]. We leveraged social support through a family member who may also have a high risk for breast cancer to promote health across the family through a digital health intervention.

### Objective

The overall objective of this study was to develop a human-centered, family dyad–based digital health intervention to promote increased movement among individuals who are cancer survivors and identify as Black and their first-degree relatives. We describe herein the development process and feasibility, usability, and acceptability results from a 4-week pilot study.

## Methods

### Study Overview

This 3-phase study presents the steps taken to develop a digital health intervention specifically for Black breast cancer survivors and their first-degree relatives (parent, child, or sibling) to help people move more and sit less, meaning we aimed to decrease sedentary time and, for some, to promote increased PA. A long-term goal of this research program is to improve family health for those at high risk for breast cancer. Therefore, for this study, the authors aimed to create family-based dyads and assess the participants’ perceptions and experiences, as well as the feasibility of this approach. This study included a design phase (phase 1) using human-centered design principles to create a working prototype of a smartphone app [23,24]. After the design phase, user-experience testing (phase 2) was completed. The study ended with a single-arm pilot study conducted to evaluate the feasibility, usability, and acceptability of both the app and the study protocol (phase 3).

### Ethics Approval, Informed Consent, Participation, and Study Registration

The Dana-Farber Cancer Institute was the institutional review board of record for this study (#20-104), and it approved all study procedures before each phase. All participants completed informed consent. Data were deidentified for analysis and maintained on a university-encrypted server. All participants received a nominal monetary gift after study assessments. The study was registered on ClinicalTrials.gov (NCT05011279).

### Phase 1: Intervention Design and Development

#### Participants

The study team established advisors (authors KBW and SP) to engage in the study process from the beginning. These advisors helped identify community leaders to provide input on the study purpose, intervention components, recruitment methods, and branding. Community leaders self-identified as Black and led community organizations historically serving the local Boston, Massachusetts, United States, or metro area Black community. The organizations represented groups for Black breast cancer survivors, sports centers, and senior centers. The advisors made initial contact, and study investigators followed up with an informational invitation email and letter, which included consent information. Community leaders participated in multiple one-on-one and group interviews throughout the study. A US $300 gift card was provided to each community leader at the end of the study.

Community leaders facilitated the recruitment of participants through public postings and emails sent to various organizations as well as flyers. Participants identified as adult, English speaking and reading, Black, either a breast cancer survivor or a first-degree relative (parent, child, or sibling) of a breast cancer survivor, and having ever used a smartphone. Breast cancer survivors were excluded if they were currently in active cancer treatment (except for ongoing hormonal therapy).

#### Methods

Human-centered design focuses on the needs and preferences of the people using the app as opposed to designing the system without input. Thus, we involved community members in creating the app via semistructured interviews to assess their needs, contexts, and perspectives to improve engagement with the app [25,26]. The participants in our study were actively involved in giving feedback and input about the designs we presented; they were not involved in drafting mock-ups or sketches or writing of content [27].

We completed a semistructured interview with the convenience sample of community leaders and community member participants over the telephone or Zoom (Zoom Video Communications, Inc; owing to the COVID-19 pandemic) to gather feedback. We asked questions about community engagement, the use of technology, study goals, considerations of sedentary behavior, and working as a family dyad. Specifically, example questions for community leaders were geared toward understanding community engagement needs, such as “How might you recommend approaching individuals to participate in the study?” and “What do we need to know about how to approach the community with this intervention so that we can facilitate success?” In the case of community members, we wanted to understand what content would be helpful and how to engage the dyad. Therefore, example questions included “What does your typical day look like? (Probes: “When do you move most?” “When do you sit most?” and “How might you move more?”) “If you were to design a program for breast cancer survivors and their first-degree relatives to move more, what would it look like, what would it include?” and “What would it be like for you to be paired with a family member?” The interviews, which were conducted by a member of the study team and a trainee or study staff member, lasted approximately 45 minutes and were audio recorded and deidentified for analysis. Community members were offered a US $30 gift card at the end to thank them for participating. Data were thematically analyzed [28] in a rapid and applied way [29,30] to inform intervention development. Analysis was conducted using Atlas.ti software (version 9.1.7.0).

Interviews were conducted with 5 community leaders and 15 community members (n=9, 60%, breast cancer survivors and n=6, 40%, first-degree relatives) to understand perspectives about moving more and sitting less within the context of the family and cancer survivorship within Black communities.

#### Intervention Development (Working Prototype of the App)

Using the feedback from the interviews and information from the literature and theory, we created the first iteration of a working prototype of the smartphone app, MoveTogether (MT). Table 1 displays the intervention’s behavior change content guided by SCT. We aimed to develop a working prototype of the app that was easy to use, culturally relevant, acceptable, and engaging.

The culturally informed content was guided by the conceptual framework developed by Joseph et al [31]. The framework includes 3 levels of cultural considerations. Level 1 focuses on how visually appealing the features are, whether the data reflect the population being served, and whether the study staff share population characteristics. Level 2 considerations include physical appearance norms and religious norms. Level 3 incorporates the population’s collective wisdom, self-sacrifice, and experiential knowledge. The MT app and the associated web-based infographics included level 1 and level 2 cultural considerations, such as using inclusive graphics that depicted persons who are Black of all ages and body types as well as the type of activity recommendations, statistics, and information targeting Black individuals (Multimedia Appendix 1). In addition, our team of study staff, advisors, and trainees reflected the demographic characteristics of the target population and provided feedback about the appropriateness of the intervention content. Specifically, qualitative data collection and study coordination were led by individuals who are demographically representative of the target population.

The working prototype was configured using PiLR software (MEI Research Ltd), which is designed for ecological momentary assessment (EMA) and used to create high-fidelity prototype apps that are more realistic and less expensive than programming a custom app. Working prototypes are appropriate for testing concepts, interactivity, graphics, and workflows (Nielsen UX [23]). The PiLR software was created for researchers to configure their smartphone apps at a relatively low cost. The basic license used in this study is limited in its functionality, with additional features requiring programming by the company. The app is available for download in both the Apple Store (iOS) and Google Play Store (Android).

Table 2 and Multimedia Appendix 1 depict the working prototype components and screenshots. The app has two main screens: (1) a welcome screen with a study logo that fades into (2) the main screen displaying 10 icons. Users clicked on an icon to reveal subscreens. The app was developed with the potential for daily use. Users were prompted with notifications reminding them to select a move-more or sit-less daily goal from a list derived during the interviews. Participants could start by selecting either a move-more goal or a sit-less goal and were then prompted to select another goal for the goal that was not first primarily identified, such as self-reporting step counts, meeting goals (yes, no, or almost), and entering free-text comments about their day if they chose. In addition, users were prompted to engage the family member (dyad buddy) through motivational messaging and goal setting.

**Table 1:** Intervention content and components informed by social cognitive theory (SCT) to promote physical activity behaviors and reduce sedentary behaviors.

Triadic factors and constructs		Behavior change technique or strategy
**Behavioral factors**		
	Monitoring	Prompted self-monitoring
	Self-regulation and goal formation	Prompted intention formation, specific goal setting, and review of behavioral goals
**Person factors**		
	Behavioral capability and outcome expectations	Provision of educational (knowledge) and instructional materials as well as resourceful websites
	Self-efficacy	Provided small steps for mastery experience and verbal persuasion
Environmental factor: social support		Prompted dyad *buddy* to provide social support for behavior and facilitated positive, supportive communication between the dyad

**Table 2:** Description of the behavior change strategies targeted in the contents of the working prototype app (refer to Multimedia Appendix 1 for the icons used).

App icon	Behavior change strategy	Description
Welcome to MoveTogether	Instructional	Welcome message and brief description about the app
How to use the Application	Instructional	How to use the app, an infographic that provides greater detail than the *Welcome* icon
Set a goal for today	Goal setting	Survey with up to 10 questions to help set the goal for the day, depending upon whether the user wants to move more, sit less, or have a day of rest. Goal questions included identifying the activity and duration, as well as where the activity would be executed and with whom
Your Goals & Steps	Self-regulation	Summary of the goals set for the day based on goal survey and number of steps participant reported for the day
How did you do today?	Self-monitoring	Self-reporting progress with 3 questions about whether the goal or goals were met, the number of steps taken for the day, and free-text comments about the participant’s day
Message your Buddy	Social support and information	Infographics about how to support the buddy, benefits of social support, sample messages to send, and a place to send the buddy a message and to see what the buddy sent the participant
Motivational Message	Verbal persuasion	Encouraging message (generic and static) and an inspirational message (generic, changes each day)
Did you know?	Knowledge	Nine infographics about topics ranging from cancer risk factors and genetic counseling to sit-less strategies, how to set smart goals, and physical activity recommendations (generic, changes each day)
Resources	Knowledge	Links to study website with links to >25 outside websites about physical activity, nutrition, family support resources, local events, cancer risk assessment sites, and a library of culturally relevant infographics created for the study

### Phase 2: User Testing

During the user-testing phase, iterations of a working prototype app were evaluated by community members. Consented participants, who were also part of phase 1 (along with 2 additional community members meeting the same eligibility criteria), met with a research assistant over telephone or Zoom and received instructions about downloading the app to their mobile phone and logging in with their username and password. Users *did not* use the app before the testing session and were neither paired with a buddy nor given a step tracker.

The session began with using a think aloud method to test app usability, guided by a script, followed by a semistructured interview to obtain specific feedback. After listening to a brief introduction about the session’s purpose, participants viewed the app content and thought aloud about what they were seeing or doing [23,32].

Participants were prompted with questions about features and functionality, which included look and feel, notifications, graphics, layout, usability, likes and dislikes, and other reactions. Example prompts included “When you are on the home page, what were your eyes drawn to?” “Is there anything off-putting or offensive?” and “What would you like changed to make it easier for you?” Users were also asked specific questions about their impressions and experiences with the app. At the end of the session, participants were asked overall what they liked, did not like, and wished was done differently.

Consistent with iterative user-centered design methods, modifications to the app were made after every 1 or 2 testing sessions. Additional rounds of user testing were completed with the near-final working prototype to make reasonable adjustments within the project’s scope and budget. In addition, the key advisors also used the near-final prototype and provided feedback.

### Phase 3: Pilot Study

#### Overview

The refined MT app was evaluated in a 5-week, single-arm pilot study. The primary aim of the pilot study was to assess the usability and acceptability of the app and describe enrollment and completion rates in a dyad-based program. The measures described in the *Data Collection Methods* section included baseline and final-week questionnaires, the assessment of accelerometer wear data, and a poststudy interview. We also measured engagement with the app over 4 weeks. Participants were provided an activity-tracking watch to go with the app, which they could keep, and a US $100 gift card for participating in the study.

#### Participants

Inclusion criteria for the pilot study were the same as those for phase 1, with a few exceptions. Participation required consent from both a cancer survivor and a blood relative to be eligible for enrollment. Individuals had to be willing to download the smartphone app and meet on a Health Insurance Portability and Accountability Act (HIPAA)–compliant remote technology platform (Zoom) or over the telephone. Those individuals who reported being pregnant or requiring medically supervised PA were excluded. All participants were adults aged >21 years. All *buddies* were first-degree relatives of the breast cancer survivors. The study team tracked enrollment, attrition, and completion. No criteria were provided for *buddy* interaction. However, prompts and app pushes were included to facilitate discussion.

#### Data Collection Methods

Behavioral, psychosocial, and demographic questionnaires were administered on the web using the REDCap (Research Electronic Data Capture; version 12.0.19; Vanderbilt University) tool hosted at Mass General Brigham at baseline and at the end of the study. The investigators, drawing from standard measures, including the US Census Bureau, Pew Research Center Surveys, and financial toxicity measures, developed an 18-item demographic questionnaire to administer at baseline.

The System Usability Scale (SUS) [33] assessed usability with a 10-item scale, with scores ranging from 0 to 100; a score of >68 is considered above average [34]. On the basis of similar measures, the investigators developed a 15-item *questionnaire on acceptability* of app components and a 6-item *questionnaire on research study participation*. The questions are included in Multimedia Appendix 2. A final item at both time points invited open-ended feedback on the questionnaires.

Self-reported PA was measured with the 16-item Global Physical Activity Questionnaire (GPAQ) [35]. The Social Support for Exercise Survey included 13 items measuring friend and family support for PA [36]. Sedentary behavior was assessed with the 18-item Sedentary Behavior Questionnaire (SBQ) [37]. The Patient-Reported Outcomes Measurement Information System (PROMIS) Global Health Scale v1.2 included 10 health and quality-of-life items [38]. These data are outside the scope of this paper and will be reported elsewhere.

#### activPAL4

Sedentary time was measured with a small, lightweight triaxial accelerometer, the activPAL micro (PAL Technologies) [39,40]. The device has excellent validity in measuring sedentary time. Participants were instructed at baseline to wear the device on the right or left thigh, secured with Tegaderm (3M) transparent film dressing, 24 hours a day for 1 week at baseline (before the intervention) and during week 4 (last week of the intervention). The device could be removed for a short time if needed. The devices were initiated and then mailed to the participants’ homes using express or overnight delivery. The research assistant met the participants via Zoom after delivery to review the wear protocol, answer questions, record the first day of activPAL wear, and generally support the participants. The participants mailed the device back to the study team in a return package after 1 week; the devices were sent again to the participants to be worn during week 4 of the intervention. Data were analyzed using PAL Software Suite (version 8.11.8.75; CREA algorithm version 1.3).

#### End-of-Study Interviews

All participants were invited to participate in a brief 30-minute telephone interview at the end of the study to describe their experience with the intervention, wearable device, and study assessments. A semistructured interview guide was used (Multimedia Appendix 2). A study investigator and a trainee conducted the interviews, audio recorded them, and took field notes.

#### App Engagement

Participant log-in, goal setting, and messaging were tracked through the MT app.

#### Data Analysis

All quantitative data were summarized using descriptive statistics (means, medians, and frequencies) using SPSS software (version 28.0; IBM Corp).

The feasibility of the study protocol was described by summarizing the recruitment, measurement, retention, and acceptability of the measurement protocol. We aimed for a retention and measurement rate of ≥70% (at least 70% of the participants would complete baseline and postassessment measures).

Acceptable engagement with the MT app was defined as use of any app feature 4 out of 7 days at a minimum.

The usability of the app was determined by an SUS score of >68. The acceptability of the app was assessed with a quantitative survey. We aimed for acceptable or positive ratings from 75% of the participants within each domain (useful, helpful, or acceptable). The acceptability of the app was also evaluated qualitatively.

The feasibility of wearing the activPAL monitor for a 7-day protocol was determined by setting a criterion of 10 hours of valid wear time per day as captured by the device. We evaluated the feasibility of mailing the device to participants’ homes with instructions and a follow-up telephone call to answer their questions. The primary purpose was to report the percentage of participants who completed the protocol to understand adherence and feasibility of the method. An adherence of >70% of the study participants to the protocol was deemed feasible.

The end-of-study interview audio recordings and field notes were thematically analyzed by the study team, study staff, and trainees for relevant themes or content.

## Results

### Phase 1

The qualitative findings from this phase are briefly summarized herein. The majority of community member participants were aged >50 years (11/15, 73%; mean age 56.5, SD 13.4 years); female (14/15, 93%); not married (13/15, 87%); had at least some college education or more (12/15, 80%); reported being non-Hispanic (8/10, 80%); and reported a great deal of difficulty paying bills (10/15, 67%), with 40% (6/15) of the participants reporting that they took less medication than prescribed owing to cost. Overall, participants responded favorably to an intervention focused on reducing sedentary behaviors. Sedentary behavior was considered more accessible than PA to individuals, particularly those with low motivation to be physically active or those with comorbid health conditions. Participants discussed motivating factors for being less sedentary, including partnering and connecting with others. Participants felt that they would be more accountable if they had a partner who possessed positive qualities, such as motivation, for them to maintain their goals. In addition, they also conveyed that it would be more enjoyable to participate in MT with another person. Some shared that they were open to partnering with a family member, whereas others expressed interest in partnering with another cancer survivor or peer. Positive feedback provided as part of the study was stated to help motivate the individual to be more active, such as receiving a congratulatory SMS text message from a partner when they achieved the activity goal. Using technology with a partner to engage in a PA-focused intervention was acceptable to the participants. Participants shared that coaching and support regarding how best to use the technology would be necessary, especially for those who may not be very familiar with using mobile apps. Participants provided feedback on the name of the intervention and suggested ways to come up with a name that users would find motivating. The findings, along with the literature and theory, informed app development.

### Phase 2

Six community members (users), 4 (67%) of whom participated in phase 1, completed user-experience testing. Overall, participants responded favorably to the app’s display of motivational or inspirational quotations and inclusion of *buddies* for accountability or as a support network. The feedback was used to make iterative changes to the app within the scope of the project and limitations of the prototype software’s functionality. These included creating a feature of *buddy* messaging within the app; improving the icons’ graphics, appearance, and layout; and modifying exemplars of activity. Some suggestions, such as incorporating stories, podcasts, message boards, music, and competitions, will be implemented in a future study. Participants also wanted to see the progress made by their *buddy* and to have the app sync to activity-monitoring technology and display results within the app. Of the 6 users, 1 (17%) felt that the prototype was “simplistic,” and 2 (33%) were concerned about data privacy.

### Phase 3

#### Participant Characteristics

Table 3 displays the characteristics of phase 3 participants (pilot study; n=10). All participants reported being born and completing their education in the United States. Regarding difficulty paying bills, of the 10 participants, 2 (20%) reported some difficulty, 1 (10%) reported quite a bit of difficulty, and 1 (10%) reported a great deal of difficulty. The majority of the participants reported that they used a smartphone *very often* (6/10, 60%), followed by *often* (3/10, 30%), and *sometimes* (1/10, 10%); and 90% (9/10) reported that they had used a health app.

**Table 3:** Characteristics of participants in phase 3 (pilot study; n=10).

Characteristics		Values
Age (years), mean (SD)		45.9 (15.69)
**Age (years), n (%)**		
	<30	1 (10)
	30-50	6 (60)
	>50	3 (30)
Sex (female), n (%)		10 (100)
Breast cancer status: survivor, n (%)		5 (50)
**Marital status, n (%)**		
	Single	7 (70)
	Married or partnered	2 (20)
	No answer	1 (10)
**Education, n (%)**		
	High school or GED^a^	4 (40)
	Some college	1 (10)
	College graduate	5 (50)
**Ethnicity, n (%)**		
	Hispanic	2 (20)
	Non-Hispanic	8 (80)
**Reported difficulty paying bills, n (%)**		
	A great deal	1 (10)
	Quite a bit	1 (10)
	Some	2 (20)
	A little	3 (30)
	No difficulty	3 (30)

^a^GED: General Educational Development Test.

#### Feasibility of the Study Protocol

##### Enrollment and Attrition

Of the 22 eligible individuals who responded to recruitment materials for phase 3 (pilot study), 10 (45%) consented and enrolled over the 3-month recruitment phase. Only 1 (10%) of the 10 participants had taken part in a previous study phase (user testing). In phase 3, all participants completed the entire 5-week study.

##### Questionnaires

Of the 5 dyads recruited, all completed the baseline and poststudy questionnaires. The mean SUS score was 72 (SD 14.3), which met our threshold to deem the intervention usable. The lowest scoring usability items were *like to use the app frequently* (median score of 3) and *cumbersome* (median score of 3).

#### Feasibility of Wearing the activPAL Monitor

Participants were asked to wear the device for 7 days during baseline assessment and for 7 days during the last week of the study (a total of 14 days). Multimedia Appendix 3 displays activPAL wear times and activity data. Of the 14 days, 60% (6/10) of the participants included a valid, 10-hour wear time of ≥4 days. Of those days where data were not valid, 2 (20%) of the 10 participants reported following the wear protocol, but no valid data were detected. In addition, 2 (20%) of the 10 participants reported issues at baseline and postintervention data collection days. Generally, participants reported that the activPAL monitor was easy to wear. Of the 10 participants, 1 (10%) noted that it got in the way during strengthening exercises, 1 (10%) wanted a stronger adhesive tape, and 1 (10%) indicated difficulty wearing the device when going through airport security.

#### Usability and Use of the MT App

The app was found helpful by 70% (7/10) of the participants, the *buddy* feature was found useful by 90% (9/10), and the Garmin step-tracking watch was found useful by all participants. Overall, the study was viewed favorably, with 90% (9/10) stating that they would recommend the app to others, whereas all participants would recommend others to participate. Table 4 summarizes acceptability data.

**Table 4:** Acceptability and utility of the MoveTogether app (n=10)^a^.

Items^b^	Agree or strongly agree, n (%)	Values, median (range)	Prefer not to answer, n (%)
Was useful	7 (70)	4 (1-5)	0
Gave me information I can trust	5 (50)	5 (3-5)	1
Was judgmental	0 (0)	1.5 (1-2)	0
Was acceptable to me	8 (80)	4 (3-5)	1
Was not helpful for me	2 (20)	2 (1-5)	0
Seemed like it was written for me	2 (20)	3 (1-5)	0
Did not speak to me	2 (20)	2 (1-4)	2
Gave me new ideas for moving more	7 (70)	4 (1-5)	0
Gave me new ideas about sitting less	8 (80)	4 (1-5)	0
Helped me understand the importance of moving more	9 (90)	4.5 (2-5)	0
Helped me understand the importance of sitting less	8 (80)	4.5 (2-5)	0
Encouraged me to try something new	8 (80)	5 (2-5)	1
Made me think about my buddy’s health habits	6 (60)	5 (2-5)	1
Helped me think of new ways to help my buddy be active	7 (70)	4 (2-5)	1
Gave me new ideas for how to support my buddy	7 (70)	4 (2-5)	0

^a^ Missing data not included in the statistics.

^b^Scale for items: 1=strongly disagree, 2=disagree, 3=neither agree nor disagree, 4=agree, and 5=strongly agree; participants could also choose prefer not to answer.

Feasibility of engagement was set to at least 4 times per week over the 4-week (28-day) period. Table 5 displays the total number of days a dyad submitted at least 1 survey on the app, which indicates active use for the day. The average number of days was 20.2 for survivors and 18.6 for first-degree relatives. Of the 10 participants, 7 (70%) used the app at least 4 times per week, meeting our criterion for app feasibility.

The most frequently used features were setting a goal and reporting on steps and goals for the day, followed by sending a message to one’s *buddy*. Table 5 shows the number of buddy messages sent per dyad. Although we did not require users to send a message to their *buddy*, the app prompted users to send messages, and all participants sent at least 1 message throughout the study. There were 106 messages sent, with survivors sending 8.6 and first-degree relatives sending 12.6 messages on average. Assuming 280 possible days (10 users × 28 days), *buddy* messages were sent on 106 (37.9%) days.

In total, 66 move-more goals were submitted. Goals included *walking or hiking* (36/66, 55%), *strengthening exercises* (10/66, 15%), *my favorite activity* (6/66, 9%), *aerobics or fitness* (6/66, 9%), *yoga or stretching* (5/66, 8%), and *running or swimming* (3/66, 5%). In total, 58 sit-less goals were set (n=15, 26%, initial goals and n=43, 74%, secondary goals after prompting). Of the 58 goals, 28 (48%) were to sit less at home, and 30 (52%) were to sit less at work. The most endorsed strategies for meeting the sit-less goals by setting (home or work) are presented in Table 6.

**Table 5:** Detailed use data by dyad during the 4-week intervention period (n=10 participants)^a^.

	Number of days the app was used^b^ (n=28), n (%)		Self-reported step entries (n=28), n (%)		Average step counts for days reported (SD)			Goals reported at the end of the day (n=28), n (%)			Number of text entries during the 28 days			Number of *Buddy* messages sent during the 28 days	
	S^c^	R^d^	S	R	S	R	S		R	S		R	S		R
Dyad 1	21 (75)	12 (43)	15 (54)	9 (32)	2290 (1521)	4508 (993)	15 (54)		9 (32)	0		7	4		2
Dyad 2	24 (86)	24 (86)	20 (71)	22 (79)	7807 (2310)	6797 (2411)	20 (71)		22 (79)	5		11	10		4
Dyad 3	28 (100)	24 (86)	28 (100)	22 (79)	3324 (1642)	5800 (2339)	28 (100)		22 (79)	7		4	17		18
Dyad 4	23 (82)	26 (93)	19 (68)	26 (93)	6335 (2132)	3322 (2082)	19 (68)		26 (93)	3		26	11		36
Dyad 5^e^	5 (18)	7 (25)	5 (18)	4 (14)	3612 (1608)	9209 (1673)	5 (18)		4 (14)	1		3	1		3

^a^Number of days the app was used, survivor: mean 20.2 (SD 8.9), relative: mean 18.6 (SD 8.5); self-reported step entries, survivor: mean 17.6 (SD 8.7), relative: mean 16.6 (SD 9.5); average step counts for days reported (survivor): 4673.6 (SD 2302), average step counts for days reported (relative): 5927.2 (SD 2255); goals reported at the end of the day, survivor: 17.6 (SD 8.7), relative: 16.6 (SD 9.5); number of text entries during the 28 days, survivor: 3.2 (SD 2.9), relative: 10.2 (SD 9.4); number of *Buddy* messages sent during the 28 days, survivor: 8.6 (SD 6.3), relative: 12.6 (SD 14.6).

^b^The total number of days the app was used was determined by the *surveys submitted*, which means the user did something on the app that required a response, such as setting a goal, messaging a *buddy*, or tracking daily steps.

^c^S: survivor.

^d^R: relative.

^e^The intervention period included a winter holiday.

**Table 6:** Most commonly selected strategies to meet sit-less goals specific to home or work setting (n=58 strategies).

Strategy	Home, n (%)	Work, n (%)
Set a timer on my phone to remind me to take a movement break (n=15)	7 (47)	8 (53)
Stand up rather than sit when I talk on the phone (n=14)	4 (29)	10 (71)
Take a 5-minute walking break at least 3 times during the day (n=13)	8 (62)	5 (38)
Walk down the hall to talk to a coworker rather than calling (n=4)	N/A^a^	4 (100)
Park my car farther away from the door (n=3)	N/A	3 (100)
Stand up and stretch during TV ads (n=5)	5 (100)	N/A
Walk to a local event (eg, church or farmers’ market; n=4)	4 (100)	N/A

^a^N/A: not applicable.

### Summary of the End-of-Study Interviews

All 10 participants participated in an end-of-study interview, and their responses suggest that the app was easy to use and helpful. Participants expressed appreciation for the research assistant, who was responsible for coordinating the enrollment of the dyads, sending and receiving the activPAL monitors, supporting participants in downloading the MT app, scheduling exit interviews, and sending links for the REDCap questionnaires. A participant commented that it was good to “know someone cares.” A few of the participants noted awareness that the app was made specifically for people who are Black, and 1 (10%) of the 10 participants noted that the images in the app “looked like them.”

There were very few negative comments about wearing the activPAL monitor for 7 days during baseline assessment and for 7 days during the last week of the study. Most of the relative participants mentioned that the questionnaire was “too long” and that the incentives (US $100 plus a Garmin step-tracking watch) were insufficient. The participants reported that it was challenging to complete the study survey on a mobile device. Overall, participants responded favorably to the app, found it usable and acceptable, and provided constructive feedback for future development.

## Discussion

### Brief Summary of Main Study Findings

This project aimed to engage community members in designing and developing an app to support Black breast cancer survivors and their first-degree relatives to move more and sit less. We developed an initial working prototype of the MT app and assessed its usability and study protocol. In summary, there is evidence for the usability and acceptability of the prototype app.

The use rate of the MT app is comparable to that in other PA app studies. Direito et al [41] found that only 31% of their sample accessed a PA app the recommended 3 times per week. In the study by Luhanga et al [42], only 63% of their sample accessed their app on a daily basis. Joseph et al [43] found that only 47% of their sample used their smartphone app ≥7 times per week, with the majority using the tracking function <7 times per week [43]. Similar digital health studies found lower adherence rates, for example, Poort et al [44] had only 9% of their sample access the app the recommended number of times. The literature suggests a positive correlation between use rates and health behaviors and health outcomes [45,46]; therefore, continued work that focuses on use is important.

The app was developed for Black breast cancer survivors and their first-degree relatives. Content and images were culturally informed, which is essential to improve the effectiveness of the intervention [47] and a component lacking in many publicly available cancer education app [48]. However, participants in the pilot study did not report awareness of the app being created for Black users. The features of inclusion that were part of the MT app were guided by a conceptual framework for developing PA programs for African American or Black women, as described by Joseph et al [31]. At this phase, only level 1 and level 2 considerations were included. Additional level 3 considerations, such as promoting a larger-scale ethics of care, engaging community members in delivering the intervention, and engaging faith-based programming, could build on the impact of this work and other mobile health apps [31].

In the initial design and development phase, we received positive feedback about creating a dyad-based intervention, which is consistent with the finding in the literature that social support plays a key role in the promotion of health-promoting interventions [49]. Some of the survivors had wanted to be connected with other survivors or a friend, not a relative. However, at study completion, all 5 family dyads had successfully used the app, and connecting with a relative was viewed favorably. Social connection is a hallmark concept of SCT, and there is an abundance of evidence suggest that social support is critical for promoting PA [21], including for Black individuals [22,50]. Less is known about social support for reducing sedentary time. This study represents one of the first known attempts to pair a Black cancer survivor with a first-degree relative to support each other in sitting less and moving more. By engaging in a dyad, the participants felt motivated to engage in, and respond to, messages and content in the app. It is known that many health apps are underused once downloaded [51]. Engaging a dyad might be one way to overcome this underuse and is also tied to the cultural considerations framework that advises developers to focus on kinship and social relationships to deliver health programs [31].

Through our development phase, we learned important information that can be applied in future research and implementation. We recognized the need for participant support in using all aspects of the study components and technology. Creating simple, easy-to-use, and engaging instructions facilitated success, which is consistent with recommendations in the literature [31]. In addition, having a human connection—although the project was technology based and conducted remotely—was desirable to help the participant feel connected to the study team.

### Limitations

Our study is limited to participants from 1 geographic area. Therefore, the results may not be transferable to other populations. In addition, we had missing activPAL data, despite evidence in the literature that research participants will wear accelerometers and activPAL monitors [52]; the timing of data collection for 2 (20%) of the 10 participants during the holiday season may have been a contributory factor. However, these glitches were resolved. This study was not designed or powered to examine pre-post differences in the primary measure of moving more and sitting less.

We intended to pilot the measurement protocol to determine whether it was feasible. The REDCap questionnaire administration was feasible with this sample, and we expect that modifying the activPAL protocol will result in a more significant percentage of participants with valid data.

### Conclusions

The MT app and related components were acceptable for promoting moving more and sitting less in dyads of breast cancer survivors and their first-degree relatives. The human-centered approach, which involved engaging community members in the development, is a model for future technology development work. Future work should be done to further develop the intervention based on the findings and then test its efficacy to improve sedentary behavior while considering culturally informed strategies for adoption and implementation within the community.

## Appendix

Multimedia Appendix 1 Screenshots from the working prototype of a smartphone app for Android and iOS devices for promoting moving more and sitting less in Black breast cancer survivors and their first-degree relatives.

Multimedia Appendix 2 Phase 3 interview guide.

Multimedia Appendix 3 Summary of activPAL wear times and average daily activity by participant.

## Abbreviations

EMA: ecological momentary assessment
GPAQ: Global Physical Activity Questionnaire
HIPAA: Health Insurance Portability and Accountability Act
MT: MoveTogether
PA: physical activity
PROMIS: Patient-Reported Outcomes Measurement Information System
SBQ: Sedentary Behavior Questionnaire
SCT: social cognitive theory
SUS: System Usability Scale

## Data Availability

The data sets generated and analyzed during this study are not publicly available but are available from the corresponding author on reasonable request.
