# Supplementary material for: Designing a Dyad-Based Digital Health Intervention to Reduce Sedentary Time in Black Breast Cancer Survivors and Their First-degree Relatives: Human-Centered Design Study
Source: JMIR Form Res. 2023 May 24;7:e43592. doi: 10.2196/43592 (PMC10248783; doi:10.2196/43592)
Supplement: Multimedia Appendix 1 [file formative_v7i1e43592_app1.pdf]

You Got This!

YOU HAVE THE POWER TO ACHIEVE GREAT THINGS!

Screen Shots from the Working Prototype of a Smartphone App for Android and iOS Devices

Prototype was configured on PiLR EMA v.2.8.1, copyright 2016,

MEI Research, LTD.

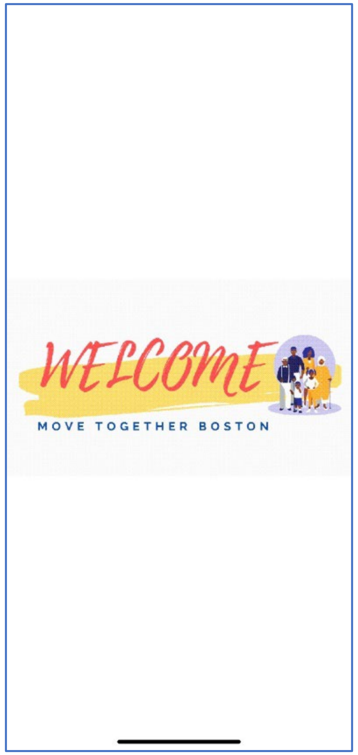
WELCOME
MOVE TOGETHER BOSTON

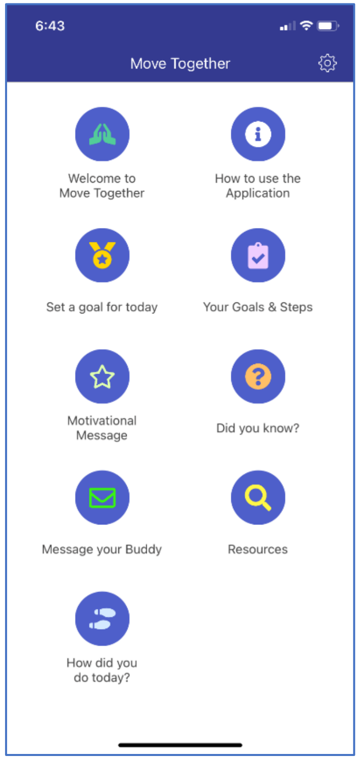
6:43
Move Together
Welcome to Move Together
How to use the Application
Set a goal for today
Your Goals & Steps
Motivational Message
Did you know?
Message your Buddy
Resources
How did you do today?

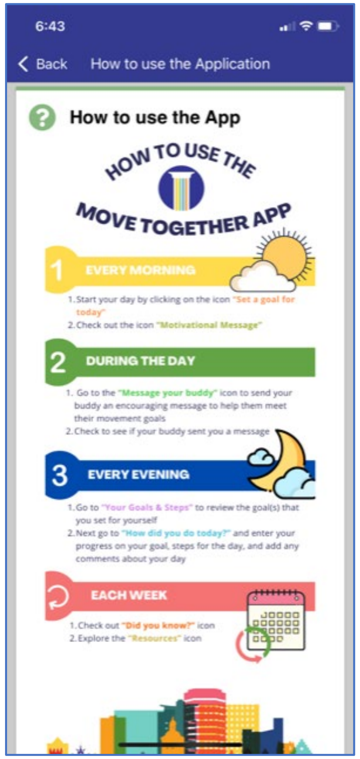
6:43
Back
How to use the Application
How to use the App
HOW TO USE THE
MOVE TOGETHER APP
1 EVERY MORNING
1. Start your day by clicking on the icon "Set a goal for today"
2. Check out the icon "Motivational Message"
2 DURING THE DAY
1. Go to the "Message your buddy" icon to send your buddy an encouraging message to help them meet their movement goals
2. Check to see if your buddy sent you a message
3 EVERY EVENING
1. Go to "Your Goals & Steps" to review the goal(s) that you set for yourself
2. Next go to "How did you do today?" and enter your progress on your goal, steps for the day, and add any comments about your day
EACH WEEK
1. Check out "Did you know?" icon
2. Explore the "Resources" icon

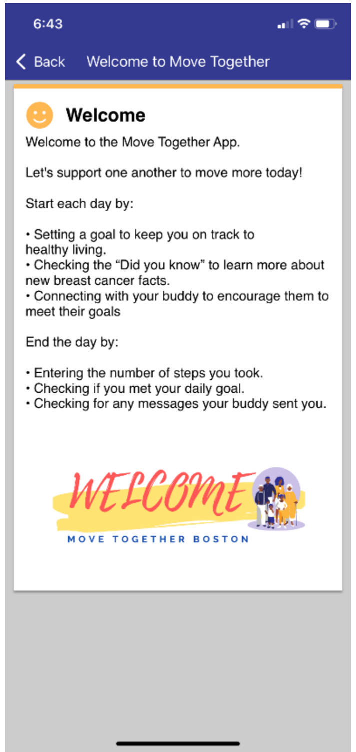
6:43
Back
Welcome to Move Together
Welcome
Welcome to the Move Together App.
Let's support one another to move more today!
Start each day by:
• Setting a goal to keep you on track to healthy living.
• Checking the "Did you know" to learn more about new breast cancer facts.
• Connecting with your buddy to encourage them to meet their goals
End the day by:
• Entering the number of steps you took.
• Checking if you met your daily goal.
• Checking for any messages your buddy sent you.
WELCOME
MOVE TOGETHER BOSTON

Homepage

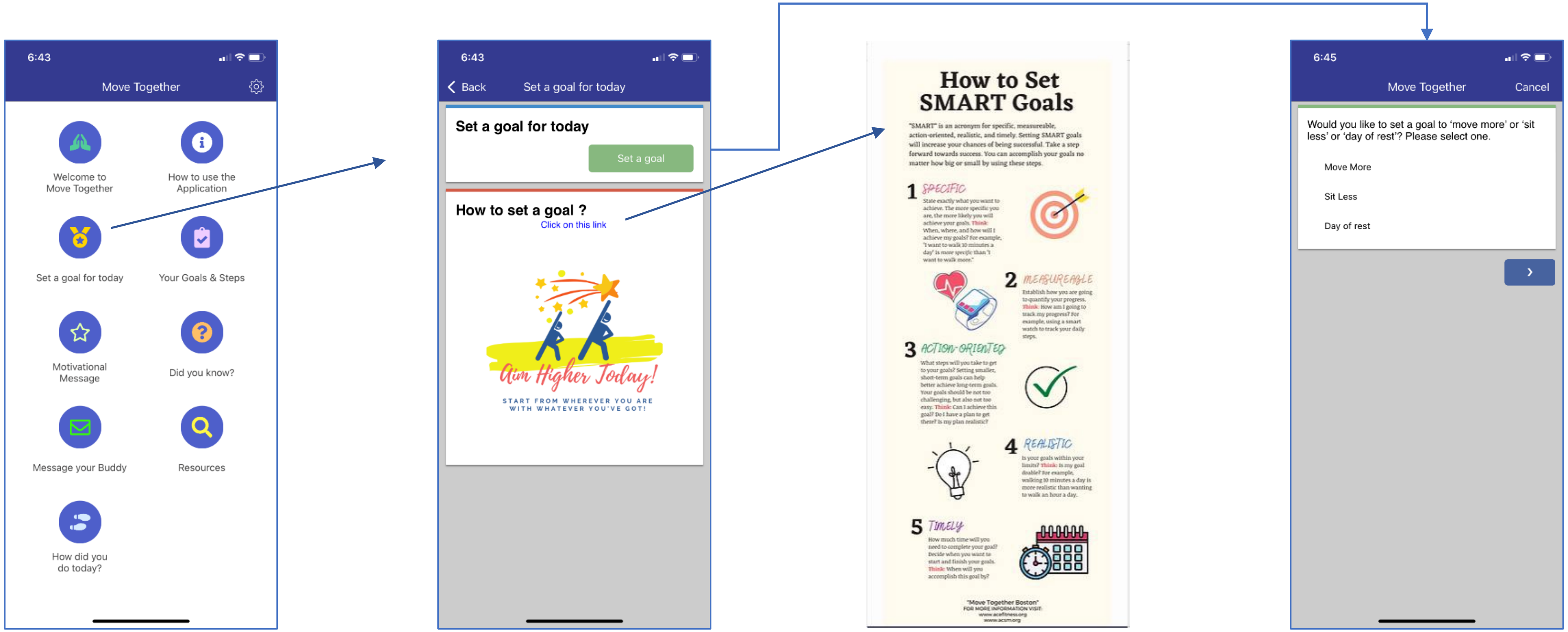

Homepage

Infographic hosted on Wix Website

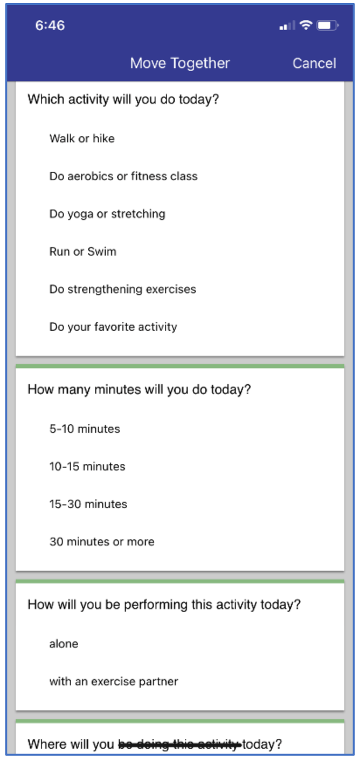

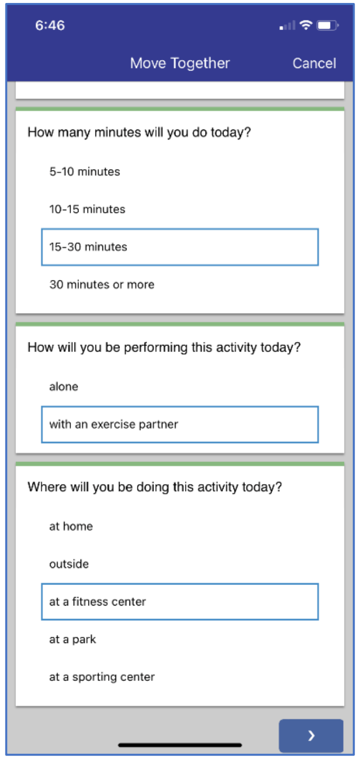

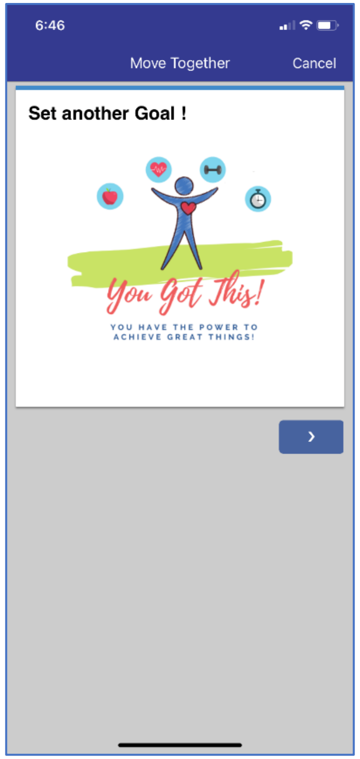

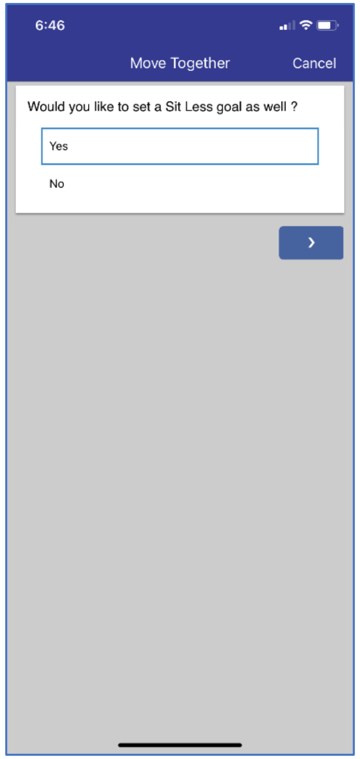

Goal Choices for Moving More

Option to set another goal

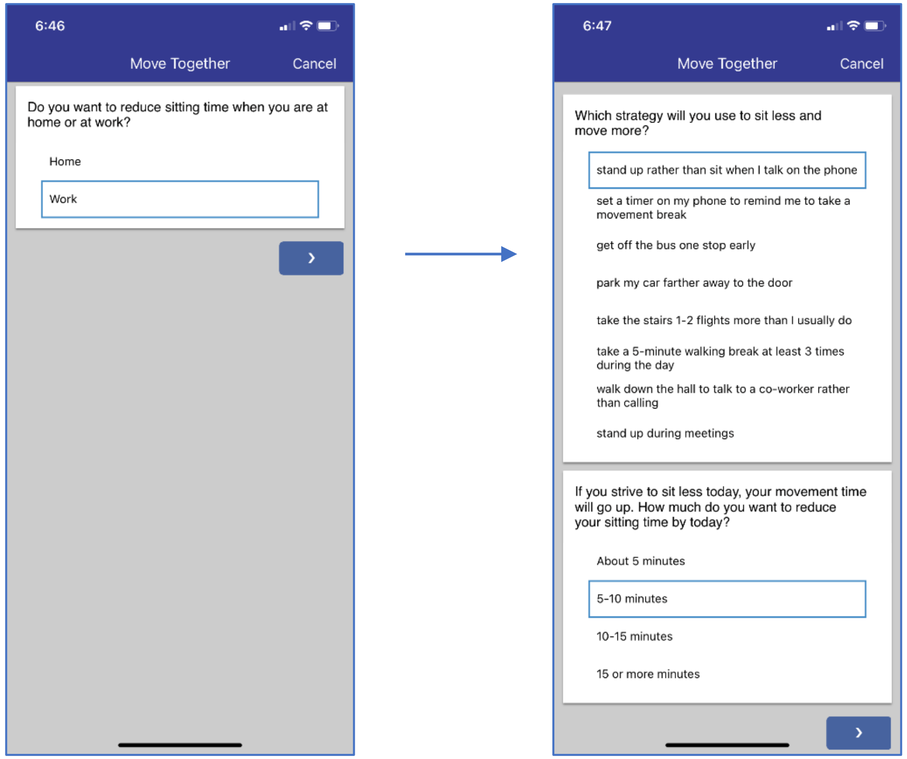

Goal Choices for
Sitting Less at Work

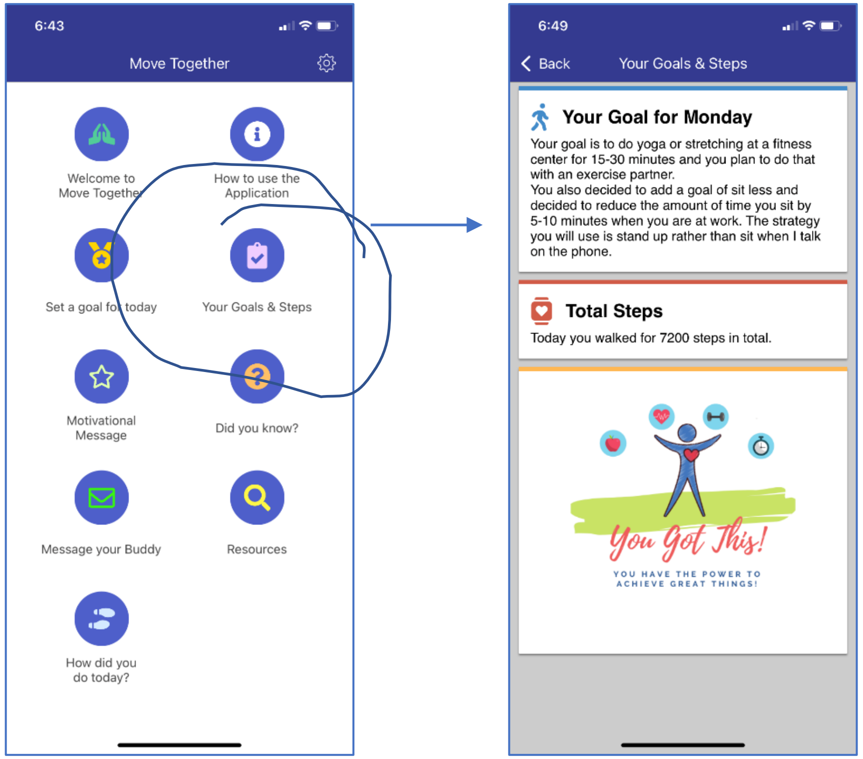

Homepage

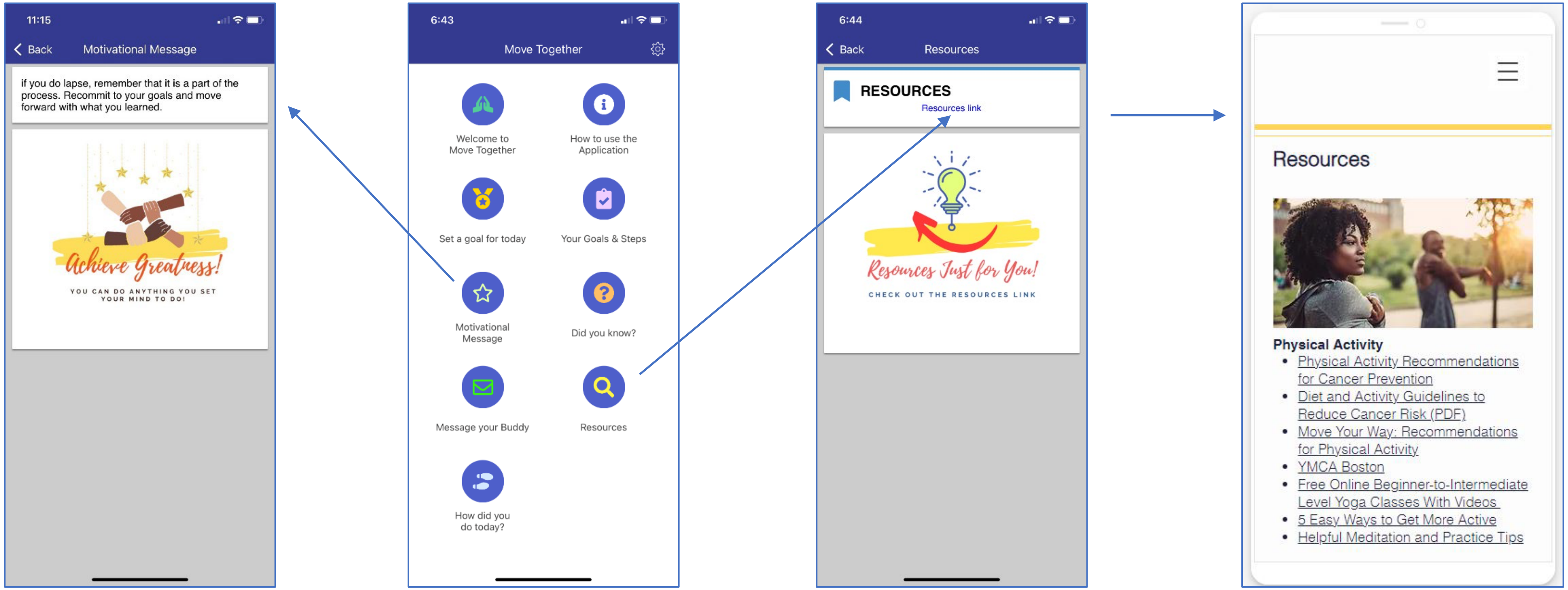

Sample web page on the resources link

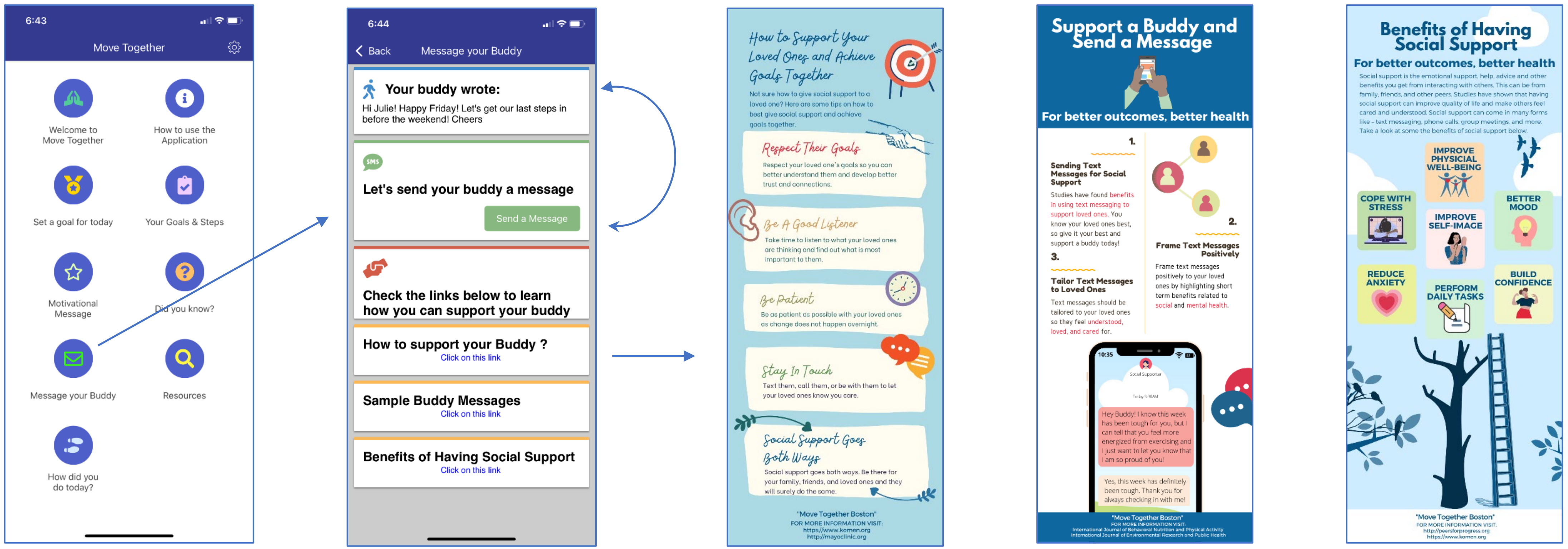
6:43
Move Together
Welcome to Move Together
How to use the Application
Set a goal for today
Your Goals & Steps
Motivational Message
Did you know?
Message your Buddy
Resources
How did you do today?
6:44
Back
Message your Buddy
Your buddy wrote:
Hi Julie! Happy Friday! Let's get our last steps in before the weekend! Cheers
SMS
Let's send your buddy a message
Send a Message
Check the links below to learn how you can support your buddy
How to support your Buddy ?
Click on this link
Sample Buddy Messages
Click on this link
Benefits of Having Social Support
Click on this link
How to Support Your Loved Ones and Achieve Goals Together
Not sure how to give social support to a loved one? Here are some tips on how to best give social support and achieve goals together.
Respect Their Goals
Respect your loved one's goals so you can better understand them and develop better trust and connections.
Be A Good Listener
Take time to listen to what your loved ones are thinking and find out what is most important to them.
Be Patient
Be as patient as possible with your loved ones as change does not happen overnight.
Stay In Touch
Text them, call them, or be with them to let your loved ones know you care.
Social Support Goes Both Ways
Social support goes both ways. Be there for your family, friends, and loved ones and they will surely do the same.
"Move Together Boston"
FOR MORE INFORMATION VISIT:
https://www.komen.org
http://mayoclinic.org
Support a Buddy and Send a Message
For better outcomes, better health
1.
Sending Text Messages for Social Support
Studies have found benefits in using text messaging to support loved ones. You know your loved ones best, so give it your best and support a buddy today!
2.
Frame Text Messages Positively
Frame text messages positively to your loved ones by highlighting short term benefits related to social and mental health.
3.
Tailor Text Messages to Loved Ones
Text messages should be tailored to your loved ones so they feel understood, loved, and cared for.
10:35
Hey Buddy! I know this week has been tough for you, but I can tell that you feel more energized from exercising and I just want to let you know that I am so proud of you!
Yes, this week has definitely been tough. Thank you for always checking in with me!
"Move Together Boston"
FOR MORE INFORMATION VISIT:
International Journal of Behavioral Nutrition and Physical Activity
International Journal of Environmental Research and Public Health
Benefits of Having Social Support
For better outcomes, better health
Social support is the emotional support, help, advice and other benefits you get from interacting with others. This can be from family, friends, and other peers. Studies have shown that having social support can improve quality of life and make others feel cared and understood. Social support can come in many forms like - text messaging, phone calls, group meetings, and more. Take a look at some the benefits of social support below.
IMPROVE PHYSICAL WELL-BEING
COPE WITH STRESS
BETTER MOOD
IMPROVE SELF-IMAGE
REDUCE ANXIETY
PERFORM DAILY TASKS
BUILD CONFIDENCE
"Move Together Boston"
FOR MORE INFORMATION VISIT:
http://peersforprogress.org
https://www.komen.org

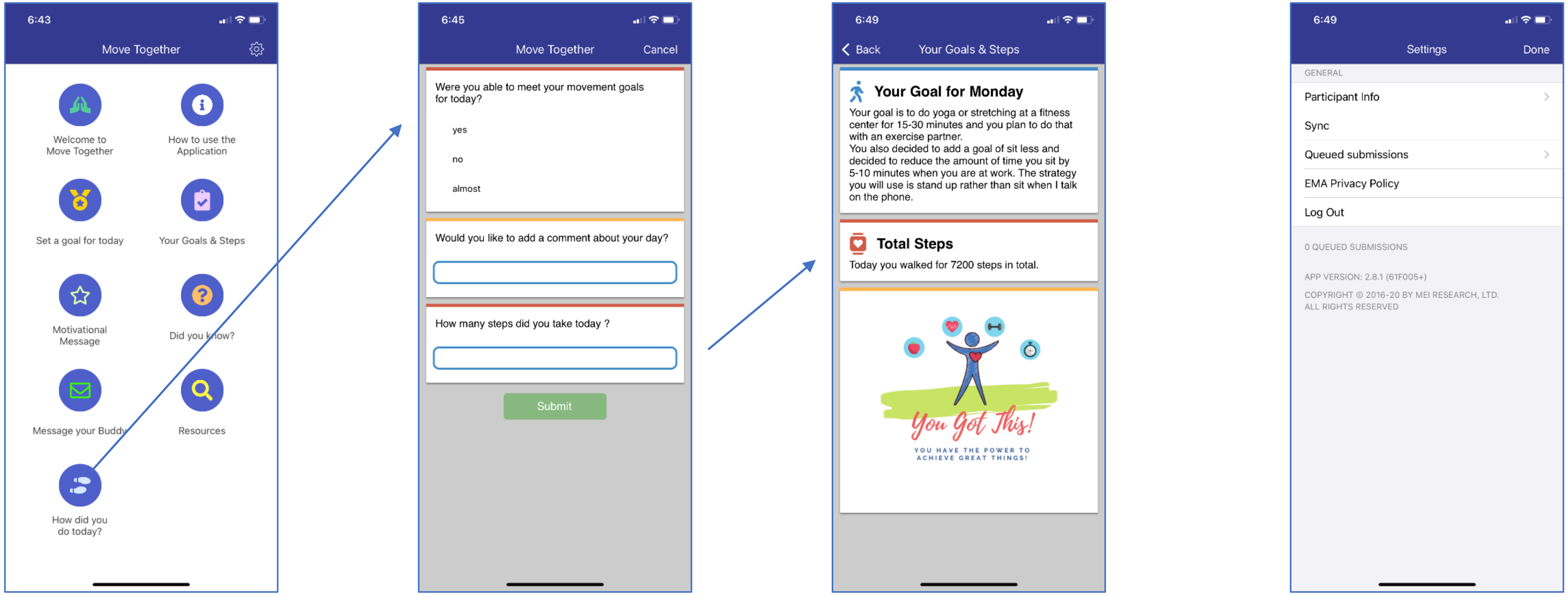

Steps entered are displayed in “Your Goals & Steps”

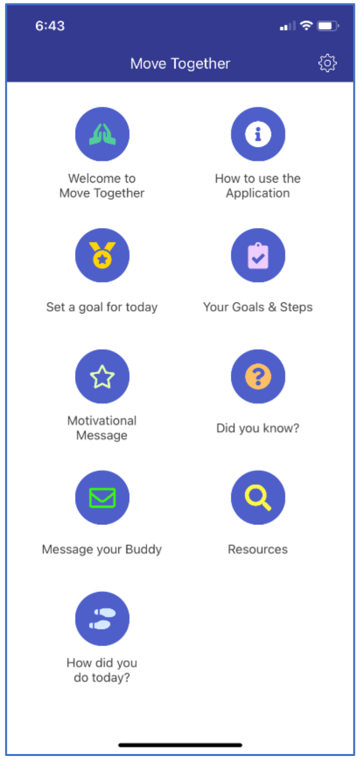

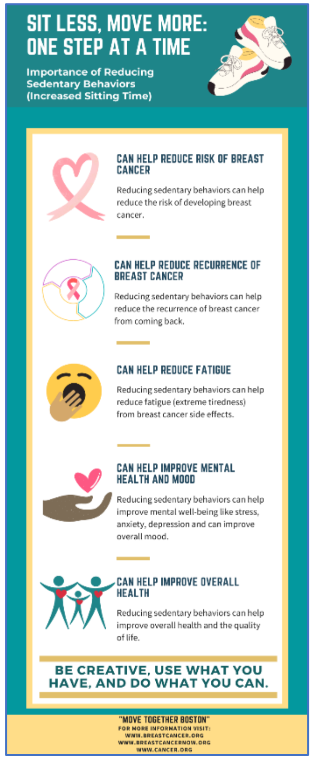

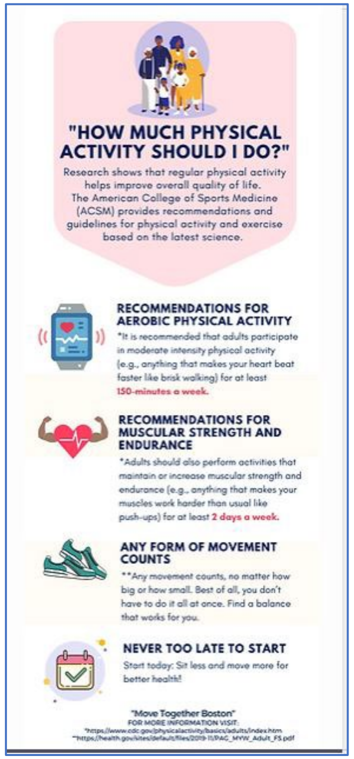

Homepage

Samples of Infographics from "Did You Know?"
