# Supplementary material for: Designing a Dyad-Based Digital Health Intervention to Reduce Sedentary Time in Black Breast Cancer Survivors and Their First-degree Relatives: Human-Centered Design Study
Source: JMIR Form Res. 2023 May 24;7:e43592. doi: 10.2196/43592 (PMC10248783; doi:10.2196/43592)
Supplement: Multimedia Appendix 2 [file formative_v7i1e43592_app2.docx]

**Phase III Exit Interview Guide**

“Thank you for agreeing to talk today about your experience taking part in the Move Together study.

“As a reminder your answers are voluntary, and you do not need to answer any questions that you do not want to answer. If you agree, we would like to audio record today’s conversation so that we can remember the details of our conversation to guide us to help others in the future. Is it okay if we get started?”

***For interviewer: indented statements are probes to be used if needed***

1. Please describe your experience overall with taking part in the study?
2. What should be done differently to improve anything?
3. What should remain the same?

***For interviewer: If not discussed in the overall question above please ask about each component aspect of the study and intervention.***

***Study onboarding***

1. Describe your experience when you first began the study.
2. How were your interactions with the study team?
3. Anything we could have done differently?
4. What were the things that most made you interested in the participating in the study to begin with?
5. Is there anything that, if it were different about the study, would have made you either more or less interested – or that you think would more attractive to future participants?
6. For example: Where you heard about it, length of time (5 weeks total), having to have a relative/partner participate, incentive gift card, keeping the Garmin watch?

***Measurement***

1. Describe your experience wearing the activPAL device to measure your movement.
2. Comfort? Convenience? Ease of instructions and use?
3. What did you think about the surveys you completed? How was it to complete the surveys?
4. Length, frequency, sensitivity?
5. Willing to do questionnaires online or telephone administered by study coordinator?

Page Break

***Intervention App***

1. What was your experience with using the Move Together app?
2. What was your experience with the website and the content on the website?
3. Were infographics understandable? Informative? What else would you want to see included?
4. The app and website are specifically meant to be used by Black or African American breast cancer survivors/relatives: What do you think about how well they did with meeting that goal?
5. What could be changed or improved to make them feel more appropriate to Black or African American survivors/relatives?
6. What was your experience with using the Garmin watch with the app?
7. Ease of instructions and use? Connected electronically or manually entered data?
8. What was your experience using the app with a “buddy”?
9. What did having a buddy add to the experience vs if you imagine just using it by yourself? Were there any drawbacks to having a buddy? Do you think you participated with the right buddy for you at this time? Why or why not? (What makes a good “buddy” for the intervention?)
10. Did you and your buddy set similar goals during the time you used the app? How do you think having similar (or different) goals to your buddy affected your experience?

***Other***

1. Before we finish, are there any other thoughts about Move Together that you would like to share with the research team?

“Thank you for taking the time to talk today. We appreciate your thoughts and participation in the research study.”
