# Supplementary material for: Designing a Dyad-Based Digital Health Intervention to Reduce Sedentary Time in Black Breast Cancer Survivors and Their First-degree Relatives: Human-Centered Design Study
Source: JMIR Form Res. 2023 May 24;7:e43592. doi: 10.2196/43592 (PMC10248783; doi:10.2196/43592)
Supplement: Multimedia Appendix 3 [file formative_v7i1e43592_app3.docx]

Table S1. Summary of activPAL wear times and average daily activity by participant.

|  |  | Time Point |  | # of Valid Days | # Week days | # Weekend Days | Avg Step Count | Avg Step Time(m) | Avg Stand Time(m) | Avg Total Sedentary Time m) | Avg Sit Time(m) | Avg Activity Score(MET.h) | Avg Number of Sit-To-Stands | Direction of change |
| --- | --- | --- | --- | --- | --- | --- | --- | --- | --- | --- | --- | --- | --- | --- |
| Dyad 1 | 1R | pre |  | **9** | 7 | 2 | **3960** | **63.59** | **482.01** | 550.78 | 391.47 | **29.60** | **34** |  |
|  | 1R | post |  | 2 | 2 | 0 | 908 | 18.74 | 360.24 | **549.78** | **347.64** | 22.37 | 23 | sat less |
|  | 1S | pre |  | **8** | 6 | 2 | 2054 | 34.07 | 331.42 | 444.91 | 296.73 | 29.35 | 29 |  |
|  | 1S | post |  | 0 | 0 | 0 | 0 | 0 | 0 | 0 | 0 | 0 | 0 | No valid data at post |
|  | 2R | pre |  | 0 | 0 | 0 | 0 | 0 | 0 | 0 | 0 | 0 | 0 |  |
| Dyad 2 | 2R | post |  | 0 | 0 | 0 | 0 | 0 | 0 | 0 | 0 | 0 | 0 | No valid data at pre or post |
|  | 2S | pre |  | 6 | 4 | 2 | **9137** | **114.47** | **295.41** | **459.26** | **342.03** | **32.89** | 38 |  |
|  | 2S | post |  | 5 | 3 | 2 | 7901 | 105.69 | 285.03 | 500.02 | 374.24 | 32.11 | **52** | No improvement |
|  | 3R | pre |  | **8** | 6 | 2 | 5336 | 69.26 | 297.38 | 572.73 | 482.15 | **31.88** | 37 |  |
| Dyad 3 | 3R | post |  | **8** | 6 | 2 | **5651** | **76.61** | **348.66** | **552.06** | **405.23** | 30.44 | **48** | moved more and sat less |
|  | 3S | pre |  | **8** | 6 | 2 | 1962 | 29.89 | 94.18 | 805.95 | 753.96 | 29.03 | 36 |  |
|  | 3S | post |  | **7** | 5 | 2 | **2270** | **34.77** | **120.84** | **550.16** | **509.76** | **29.79** | 36 | moved more and sat less |
|  | 4R | pre |  | **8** | 6 | 2 | 7164 | 105.89 | 271.67 | **511.06** | **425.92** | 31.51 | 59 |  |
| Dyad 4 | 4R | post |  | **7** | 5 | 2 | **7628** | **113.42** | **320.47** | 563.31 | 476.64 | **31.85** | **60** | moved more |
|  | 4S | pre |  | **8** | 6 | 2 | **6731** | **85.17** | **203.66** | **596.47** | **567.23** | **30.72** | **38** |  |
|  | 4S | post |  | **7** | 5 | 2 | 5500 | 73.64 | 188.32 | 653.06 | 623.953 | 30.65 | 32 | No improvement |
|  | 5R | pre |  | 0 | 0 | 0 | 0 | 0 | 0 | 0 | 0 | 0 |  |  |
| Dyad 5 | 5R | post |  | 0 | 0 | 0 | 0 | 0 | 0 | 0 | 0 | 0 |  | No valid data at pre or post |
|  | 5S | pre |  | **11** | 8 | 3 | 3876 | 59.36 | 461.11 | 431.24 | 393.05 | **30.10** | 30 |  |
|  | 5S | post |  | 4 | 2 | 2 | **4053** | **62.95** | **465.68** | **377.58** | **337.01** | 28.22 | 27 | moved more and sat less |

Note. R=relative; S=survivor; bolded numbers indicate a better profile. Averages are based on the days worn. PALanalysis software_tool_version 8.11.6.70, validation_algorithm_name is MORA, validation_algorithm_v1.0, validation_algorithm_wear_time_protocol = 10 hours, analysis_algorithm_name is CREA

analysis_algorithm_v1.3
